# Asociación de alteraciones de la función tiroidea con turnicidad/nocturnidad laboral y antecedentes de patología tiroidea en profesionales sanitarios

**DOI:** 10.23938/ASSN.1057

**Published:** 2024-04-06

**Authors:** Francisco de Borja Merino Moya, Silvia Lucena Garcia, Juan Manuel García Torrecillas

**Affiliations:** 1 Servicio Andaluz de Salud Hospital Universitario Torrecárdenas Medicina del Trabajo-Unidad de Prevención de Riesgos Laborales Almería España; 2 Servicio Andaluz de Salud Hospital Universitario Torrecárdenas Servicio de Urgencias Almería España; 3 Servicio Andaluz de Salud Unidad de Investigación Biomédica Hospital Universitario Torrecardenas Almería España; 4 Centro de Investigación Biomédica en Red de Epidemiología y Salud Pública (CIBERESP) Madrid España; 5 Instituto de Investigación Biosanitaria (ibs.GRANADA) Granada España

**Keywords:** Trabajo por turnos, Trabajo nocturno, Pruebas de función de la Tiroides, Enfermedades tiroideas, Distribución por sexo, Shift Work Schedule, Thyroid function test, Thyroid Diseases / history, Sex distribution

## Abstract

**Fundamento:**

El objetivo de este estudio es analizar la asociación entre la patologia funcional tiroidea y la exposición a turnicidad/nocturnidad laboral, y describir los trastornos tiroideos más prevalentes por turno de trabajo.

**Metodología:**

Estudio transversal realizado en un servicio de urgencias hospitalario de Almería (España). Se relacionaron los niveles de tiroxina y tirotropina (TSH) con el turno, la categoría profesional y los antecedentes de patología tiroidea.

**Resultados:**

Se incluyeron 133 trabajadores, 80,5% mujeres, edad media 46,11 años (38 a 65) y 52% personal enfermero; las mujeres mostraron más frecuentemente antecedentes tiroideos. El 81,2% trabajaba en turno rotatorio y el 11,3% en turno nocturno (12,1% de mujeres y 7,7% de hombres). El 27% mostró alteraciones tiroideas, más frecuentemente niveles elevados de TSH con niveles normales de tiroxina, especialmente en turno nocturno (61,1%). Las alteraciones de TSH fueron más frecuentes en turno nocturno que en rotatorios (53,3 vs 13,0%; p<0,001). El turno nocturno presentó valores medios de TSH en rango normal pero significativamente superiores al resto de turnos, mientras los niveles de tiroxina fueron similares. El turno diurno no presentó alteraciones. El turno nocturno y la presencia de antecedentes fueron predictores independientes de presentar alteraciones tiroideas.

**Conclusiones:**

El turno nocturno y los antecedentes de patología tiroidea fueron más frecuentes en mujeres, y ambos se asociaron con la presencia de alteraciones tiroideas, indicando la necesidad de incluir la evaluación de dichas alteraciones en los programas de vigilancia de salud y de analizar las diferencias por sexo.

## Abstract (en)

**Background:**

The study aim was to analyze the relationship between functional thyroid pathology and the exposure to work shifts / night shifts, and describe the most prevalent thyroid disorders based on the type of shift.

**Methodology:**

Cross-sectional study performed in the emergency department of a hospital in Almeria (Spain). Relationships between thyroxine and thyrotropin levels (TSH) and work shifts, professional category and history of thyroid pathology were analyzed.

**Results:**

The study included 133 workers; 80.5% female, average age was 46.11 years (38 - 65), and 52% were part of the nursing staff; thyroid disorders were more frequent in female participants. Most participants (81.2%) had rotating shifts schedules and 11.3% night shifts (12.1% female and 7.7% male). Thyroid alterations were found in 27% of the participants (usually elevated TSH levels and normal thyroxine levels), particularly in those doing night shifts (61.1%). TSH alterations were more frequent in individuals doing night shifts than in rotating shifts (53.3 vs 13.0%; p<0.001). Individuals working night shifts had mean TSH values in the normal range, although significantly higher than the individuals in the rest of the shifts; thyroxine levels were found to be similar. No thyroid disorders were found in day shift participants. Night shift and a history of thyroid pathology were independent predictors of thyroid disorders.

**Conclusions:**

Night shift schedules and history of thyroid disorders are more frequent in female, both related to the presence of thyroid disorders, indicating the need to include the evaluation of these disorders in health surveillance programs and analyze gender differences.

## INTRODUCCIÓN

La alteración de la función de la glándula tiroides es un trastorno frecuente en la población general. Un metanálisis realizado en 2014 detectó importantes variaciones en su prevalencia, probablemente atribuibles a diferencias en múltiples factores como rangos de referencia, técnicas de laboratorio, estado nutricional de yodación de la región estudiada, así como factores genéticos como raza, edad o sexo^1^. En España, los datos de prevalencia actual de trastornos de la función tiroidea se reducen a pequeñas áreas del norte, variando los resultados en función del déficit de yodo de la región estudiada^2^.

La función de la glándula tiroides consiste en producir la cantidad de hormonas triyodotironina (T3) y tiroxina (T4) necesarias para cubrir las necesidades de mantenimiento de casi todos los órganos y tejidos, especialmente el hígado, el sistema nervioso central y el cardiovascular^3^. Intervienen en el crecimiento y la diferenciación tisular, regulando numerosos procesos como el consumo de oxígeno y termogénesis, así como el metabolismo de macromoléculas^4^.

Las patologías funcionales de la tiroides pueden causar síntomas que afectan y limitan el desempeño de las tareas habituales de los profesionales durante su jornada laboral. Los síntomas más frecuentes asociados al hipotiroidismo son cansancio, intolerancia al frío, apatía e indiferencia, depresión, disminución de memoria y de la capacidad de concentración mental, aumento de peso, estreñimiento pertinaz o somnolencia excesiva^5^. El hipertiroidismo se caracteriza por nerviosismo, insomnio, palpitaciones, cansancio inexplicable, sudoración, mala tolerancia al calor, temblor de manos o pérdida de peso a pesar de coexistir con apetito aumentado o diarreas^5^.

La glándula tiroides participa, junto con el hipotálamo y la hipófisis, en un mecanismo de control por retroalimentación que regula la función tiroidea. La secreción de hormonas tiroideas está regulada por la hormona estimulante del tiroides (TSH) hipofisaria, que a su vez se regula por la hormona liberadora de tirotropina (TRH) hipotalámica. La TRH estimula la producción de TSH, glucoproteína cuya subunidad β se une a un receptor de membrana en las células foliculares, aumentando la síntesis y secreción de hormonas^2^. El eje hipotálamo-pituitario-tiroideo (HPT) detecta las variaciones en la disponibilidad de hormonas tiroideas libres y actúa mediante retroalimentación negativa para corregirlas^6^.

El sistema nervioso central controla el sistema endocrino mediante rutas neurales o humorales, o a través del ciclo sueño-vigilia. La secreción de TSH presenta una clara ritmicidad diaria relacionada con dicho ciclo y con el patrón del sueño, mientras que el HPT es controlado por el marcapasos circadiano central ubicado en el núcleo supraquiasmático hipotalámico anterior, el cual recibe información luminosa de la retina a través del tracto retino-hipotalámico y emite la señal circadiana al núcleo paraventricular hipotalámico^7^. En condiciones normales de sueño, las concentraciones plasmáticas de TSH comienzan a aumentar al final de la tarde o en las horas previas al inicio del sueño, alcanzando niveles máximos durante la primera parte de la noche. Tras alcanzar el pico nocturno de TSH, la concentración plasmática desciende progresivamente durante el período de sueño restante hasta alcanzar niveles bajos durante el día^8^. La privación del sueño y los cambios agudos del período de sueño inhiben la producción de TSH^9^^,^^10^ y tienen múltiples consecuencias metabólicas^11^.

Debido a la evolución económica, industrial y social de los países industrializados, el trabajo por turnos y los patrones de trabajo irregulares son cada vez más frecuentes, afectando aproximadamente a una quinta parte de la población trabajadora a nivel mundial^12^. El trabajo por turnos, y particularmente el nocturno, ha sido reconocido como un factor de estrés para el cuerpo humano que puede generar a largo plazo un amplio espectro de efectos negativos en la salud de los trabajadores^13^. Aunque muchas personas con trabajos nocturnos experimentan trastornos crónicos del ritmo circadiano, no abundan los estudios que muestren una relación de causalidad entre la desincronización circadiana y sus efectos adversos sobre los factores metabólicos y hormonales, en particular sobre el HPT^10^.

Por tanto, este estudio tiene como objetivo evaluar la asociación entre la exposición a turnicidad y nocturnidad laboral y las alteraciones en la funcionalidad de la glándula tiroides de trabajadores de un servicio de Urgencias hospitalario. Además, se describen las alteraciones tiroideas encontradas y su asociación con las características del personal estudiado.

## MATERIAL Y MÉTODOS

Estudio descriptivo transversal realizado en el personal de medicina y enfermería del servicio de Urgencias del Hospital Universitario Torrecárdenas (Almería), entre octubre de 2021 y febrero de 2022.

Este servicio de Urgencias se divide en dos áreas de trabajo: Urgencias Generales y Área de Observación/Sillones. Las actividades diarias y la dinámica de trabajo del personal de enfermería se distribuyen en tres tipos de turnos: un turno fijo de mañanas y tardes, un turno nocturno regular y un turno rotatorio de día y noche (también llamado anti estrés) distribuido en dos mañanas, dos tardes, una noche y tres descansos. Los facultativos de Urgencias trabajan en dos tipos de turnos: un turno regular de mañana y tarde y otro turno de trabajo consistente en dos mañanas, dos tardes, una guardia de 24 horas continuadas y tres días de descanso, siendo las funciones comunes en todos los turnos.

Se incluyeron todas las personas trabajadoras pertenecientes a las categorías profesionales de Facultativo Especialista de Área (FEA), Diplomado Universitario en Enfermería (DUE) y Técnico Auxiliar en Cuidados de Enfermería (TCAE), con al menos seis meses de antigüedad en el puesto y que aceptaran participar en el estudio mediante firma del consentimiento informado. Se excluyeron los profesionales con antecedentes de cirugía tiroidea, en tratamiento farmacológico que pudiera afectar la función tiroidea, y en situación de incapacidad laboral temporal durante el estudio.

El Servicio de Prevención de Riesgos Laborales (SPRL) efectuó el examen médico al personal sanitario como parte de la Vigilancia de la Salud^12^. Consistió en una entrevista clínico-laboral, exploración física y análisis de sangre que incluyó parámetros específicos para valorar la funcionalidad tiroidea: tirotropina (TSH) y tiroxina libre (T4). La extracción sanguínea se realizó en horario de mañana y situación de ayunas con el fin de asegurar que todo el personal incluido en el estudio estuviera en condiciones similares.

Se registraron las siguientes variables en la historia clínica-laboral de cada uno de los trabaja-dores:

- Datos socio-demográficos: sexo (mujer, hombre) y edad (años; 18-44, 45-65, ≥65);
- Categoría profesional: FEA, DUE, TCAE;
- Área de trabajo: Urgencias Generales, Observación/Sillones, ambas;
- Turno de trabajo: diurno (fijo de mañanas y tardes), nocturno (fijo de noches), y rotatorio (dos mañanas, dos tardes, una noche y tres días de descanso para DUE y TCAE; dos mañanas, dos tardes, una guardia de 24 horas continuadas y tres días de descanso para FEA);
- Funcionalidad tiroidea: concentración de TSH (rango normal: 0,38-5,33 pg/mL) y de T4 (rango normal: 0,54-1,24 ng/dL);
- Antecedentes de patología tiroidea: sí, no.

La variable de resultado fue la alteración tiroidea (sí, no) categorizada en TSH alta y T4 normal, TSH baja y T4 normal, TSH normal y T4 alta, TSH normal y T4 baja.

Los datos recogidos se registraron en una base de datos anonimizada, construida para tal fin, y procesados estadísticamente mediante los programas Microsoft Excel 2013 y Stata versión 12. No fue necesario efectuar el cálculo del tamaño muestral para obtener una muestra representativa dado que la muestra incluida coincidió con la población diana.

Las variables cualitativas se describieron mediante frecuencias y porcentajes, acompañados de su intervalo de confianza al 95%, y las cuantitativas mediante medias acompañadas de desviaciones estándar (DE) o mediana y rango intercuartílico (RIC). La relación entre el turno de trabajo y la funcionalidad tiroidea (cuyas variables TSH y T4 no presentaron distribuciones normales en cada grupo de profesionales según Kolmogorov-Smirnov) se realizó mediante la prueba no paramétrica de Kruskall Wallis para la comparación de muestras independientes (realizando U de Mann-Whitney con corrección de Bonferroni como test *post hoc*), y la prueba Chi-cuadrado (χ^2^). Se realizó un procedimiento de regresión logística binaria para evaluar la asociación de las variables estudiadas con la alteración de la función tiroidea mediante *odds ratio* y su correspondiente intervalo de confianza al 95% (IC95%). Se consideró significativo un valor de p <0,05.

El dictamen del Comité de Ética de la Investigación Provincial de Almería CEI/CEIm fue favorable y se garantizó el anonimato de los datos de filiación de los trabajadores incluidos, siendo informados previamente al examen de salud del uso de los datos exploratorios para fines de investigación. Se firmó un consentimiento informado voluntario previo.

## RESULTADOS

El SPRL del hospital realizó 149 exámenes de salud al personal FEA, DUE y TCAE del servicio de Urgencias. Se excluyeron del estudio 16 personas por los siguientes motivos: antecedentes de cirugía tiroidea (n=2); tratamiento habitual con medicamentos que pudieran afectar la función tiroidea, como amiodarona, litio o inhibidores de la tirosina quinasa (n=3); situación de incapacidad temporal durante el estudio (n=4) y renuncia voluntaria a participar (n=7; 4,7%).

Por tanto, 133 trabajadores fueron incluidos en el estudio, la mayoría mujeres (80,5%), con edad media 46,11 años (rango 38 a 65) y más de la mitad en la franja de 45 a 64 años, y DUE (52%). El 44,4% de la muestra trabajaba en Urgencias Generales y el 35,3% en el Área de Observación/Sillones. La gran mayoría trabajaba en turno rotatorio (81,2%) y solo el 11,3% en turno de noches fijas. Solo se observaron diferencias significativas por sexo para la categoría profesional: la presencia de hombres fue más frecuente entre el personal de las categorías FEA y DUE. El 10,5% del personal tenía antecedentes de enfermedad tiroidea conocida y/o tomaban levotiroxina oral; aunque los antecedentes fueron tres veces más frecuentes en mujeres que en hombres, esta diferencia no fue significativa (n=13; 12,1% frente a n=1; 3,8%; p=0,302) (Tabla 1).

**Tabla 1:** Descripción general de la población estudiada

Variable	Global	Sexo		p
	n (%)	Mujer n=107	Hombre n=26	(χ^2^)
*Sexo (mujer)*	107 (80,5)			
Edad				0,405
18-44	50 (37,6)	43 (40,2)	(26,9)	
45-64	82 (61,7)	63 (58,9)	19 (73,1)	
>=65	1 (0,8)	(0,09)		
Categoría profesional				0,003
FEA	7 (20,3)	19 (17,8)	(30,8)	
DUE	9 (51,9)	53 (49,5)	16 (61,5)	
TCAE	7 (27,8)	35 (32,7)	(7,7)	
Área de trabajo				
Urgencias Generales	59 (44,4)	49 (45,8)	10 (38,5)	
Área de Observación/Sillones	47 (35,3)	39 (36,5)	(30,8)	
Ambas	27 (20,3)	19 (17,8)	(30,8)	
Turno de trabajo				0,766
Diurno	10 (7,5)	(8,4)	(3,8)	
Nocturno	5 (11,3)	13 (12,1)	(7,7)	
Rotatorio	108 (81,2)	85 (79,4)	23 (88,5)	
Antecedentes de patología tiroidea	4 (10,5)	13 (12,1)	(3,8)	0,302

FEA: Facultativo Especialista de Área; DUE: Grado en Enfermería; TCAE: Técnico en Cuidados Auxiliares de Enfermería.

La población de estudio mostró un 27% de alteraciones tiroideas, más frecuentes de TSH que de T4 (16,5% frente a 10,5%). Los valores medios de TSH (2,2 pg/mL; DE: 1,43), y de T4 (0,85 ng/mL; DE: 0,21) se encontraban dentro de los rangos normales (Tabla 2).

El 100% de los profesionales con turno diurno presentaba niveles de TSH y T4 dentro de los parámetros de normalidad. Sin embargo, la prevalencia de alteraciones en los niveles de TSH fue mucho mayor en el turno de noches fijas (66,7%) que en los rotativos (hasta 26,8%), asociada principalmente a la mayor frecuencia de niveles de TSH alterados en el nocturno (53,3 frente a 13,4 o inferiores) (Tabla 2).

**Figura 1:**
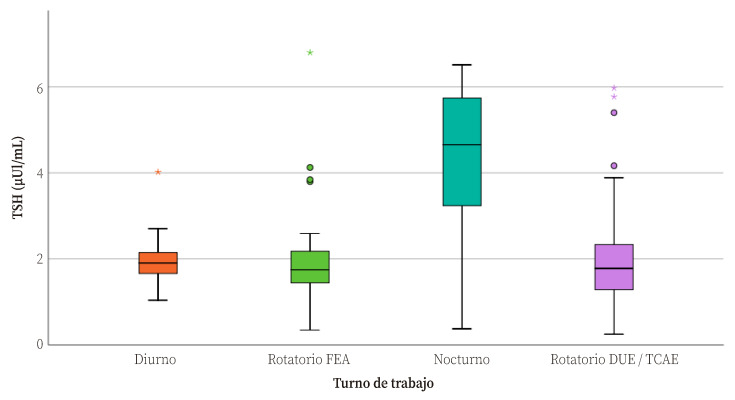
*Box Plot* de los valores de TSH en sangre por turno de trabajo.

La media de TSH en el grupo de trabajadores con noches fijas (4,25 pg/mL; DE: 1,84), en el límite superior de la normalidad en sangre, fue notablemente más alta que en el resto de turnos (p<0,001). La media de TSH en los turnos diurno y rotativo de mañanas-tardes con guardia de 24h presentaron medias muy similares (2,06 µUI/mL; DE: 0,81 y 2,07 µUI/mL; DE: 1,34 respectivamente) y superiores a la del turno rotatorio antiestrés (1,87 µUI/mL), pero de forma no significativa (p=0,103) (Tabla 2, Fig. 1). No se encontraron diferencias significativas en los niveles de T4 entre el personal de los diferentes turnos.

**Tabla 2:** Niveles de hormonas tiroideas según turno laboral

Variable	Global	Turno				p*
		Rotatorio		Diurno	Nocturno	
		DUE/TCAE	FEA (N=27)			
*TSH*						0,0001
Media (DE)	2,2 (1,43)	1,87 (1,11)	2,07 (1,34)	2,07 (0,81)	4,25 (1,84)	
IC95%	1,95-2,44	1,63-2,12	1,52-2,60	1,49-2,65	3,23-5,27	
% alterado	22,0 (16,5)	11,0 (13,4)	3,00 (11,5)	0	8,00 (53,3)	
*Tiroxina (T4)*						0,342
Media (DE)	0,85 (0,21)	0,87 (0,22)	0,84 (0,18)	0,81 (0,12)	0,78 (0,27)	
IC95%	0,81-0,89	0,82-0,92	0,77-0,91	0,73-0,89	0,63-0,93	
% alterado	14,00 (10,5)	11,00 (13,4)	1,00 (3,8)	0	2,00 (13,3)	
Alteración tiroidea (%)	36,00 (27,0)	22,00 (26,8)	4,00 (15,4)	0	10,00 (66,7)	0,0004 (χ^2^)

*: Kruskal-Wallis, con U de Mann-Whitney y corrección de Bonferroni como test *post hoc*; TSH: tirotropina; DE: desviación estándar; IC: intervalo de confianza; DUE: Grado en Enfermería; TCAE: Técnico en Cuidados Auxiliares de Enfermería; FEA: Facultativo Especialista de Área; turno rotatorio DUE/TCAE: dos mañanas, dos tardes, una noche y tres días de descanso; turno rotatorio FEA: dos mañanas, dos tardes, guardia de 24 horas y tres días de descanso.

TSH: tirotropina; Diurno: mañanas/tardes; Rotatorio FEA: dos mañanas, dos tardes, guardia de 24 horas y tres días de descanso; FEA: Facultativo Especialista de Área; Rotatorio DUE/TCAE: dos mañanas, dos tardes, una noche y tres días de descanso; DUE: Grado en Enfermería; TCAE: Técnico en Cuidados Auxiliares de Enfermería. Las cajas representan el cuartil 1 (Q1) y el cuartil 3 (Q3), y la línea la mediana; los bigotes representan desviaciones de 1,5RIC respecto al cuartil correspondiente; círculos: *ouliers* (> Mediana±1,5RIC); estrella: extreme outlier (> Mediana±3RIC); RIC: rango intercuartílico: Q_3_-Q_1_.

Respecto a los tipos de alteraciones tiroideas observadas, las más frecuentes fueron los niveles de TSH alterados con niveles de tiroxina dentro de la normalidad (n=22; 61,1%), con la misma frecuencia de niveles de TSH elevados o disminuidos. La frecuencia de niveles normales de TSH con niveles elevados o disminuidos de tiroxina fue similar (Tabla 3). El tipo de alteración se relacionó con el turno (los niveles elevados de TSH con niveles normales de tiroxina predominaron en el turno de noches fijas) y con tener antecedentes de patología tiroidea (más de la mitad del personal con antecedentes y con alteraciones, presentó niveles elevados de tiroxina con niveles normales de TSH). Como se muestra en la tabla 1, las mujeres presentan mayor frecuencia de antecedentes de patología tiroidea y de trabajo en turno de noches fijas.

**Tabla 3:** Prevalencia de tipos de alteración de la función tiroidea por turnos de trabajo

Variable	Función tiroidea alterada n (%)	p (χ^2^)	Tipo de alteración n (%)				p (χ^2^)
			T4 normal		TSH normal		
			TSH alta	TSH baja	T4 alta	T4 baja	
Edad							
18-44	12 (24,0)	0,6701	3 (25,0)	3 (25,0)	4 (33,3)	2 (16,7)	0,8641
45-64	24 (29,3)		8 (33,3)	8 (33,3)	4 (16,7)	4 (16,7)	
>= 65	0		0	0	0	0	
Sexo							
Hombre	4 (15,4)	0,3542	1 (25,0)	1 (25,0)	1 (25,0)	1 (25,0)	0,8249
Mujer	32 (29,9)		10 (31,3)	10 (31,3)	5 (15,6)	7 (21,9)	
Turno							
Diurno	0	0,0004	0	0	0	0	0,0008
Nocturno	10 (66,7)		7 (70,0)	1 (10,0)	1 (10,0)	1 (10,0)	
Rotatorio	26 (24,1)		4 (15,4)	10 (38,5)	5 (19,2)	7 (26,9)	
Categoría							
DUE	21 (30,4)	0,2749	5 (23,8)	5 (23,8)	5 (23,8)	6 (28,6)	0,6353
FEA	4 (18,8)		1 (25,0)	2 (50,0)	0	1 (25,0)	
TCAE	11 (29,7)		5 (45,4)	4 (36,4)	1 (9,1)	1 (9,1)	
Antecendentes de patología tiroidea							
No	21 (21,0)	<0,0001	(33,3)	(28,6)	2 (9,5)	6 (28,6)	<0,0001
Sí	11 (78,6)		(36,4)	(9,2)	6 (54,4)	0	
Global	36 (27,0)		11 (30,6)	11 (30,6)	8 (22,0)	6 (16,7)	

TSH: tirotropina; T4: tiroxina libre; DUE: Grado en Enfermería; TCAE: Técnico en Cuidados Auxiliares de Enfermería; FEA: Facultativo Especialista de Área.

**Figura 2:**
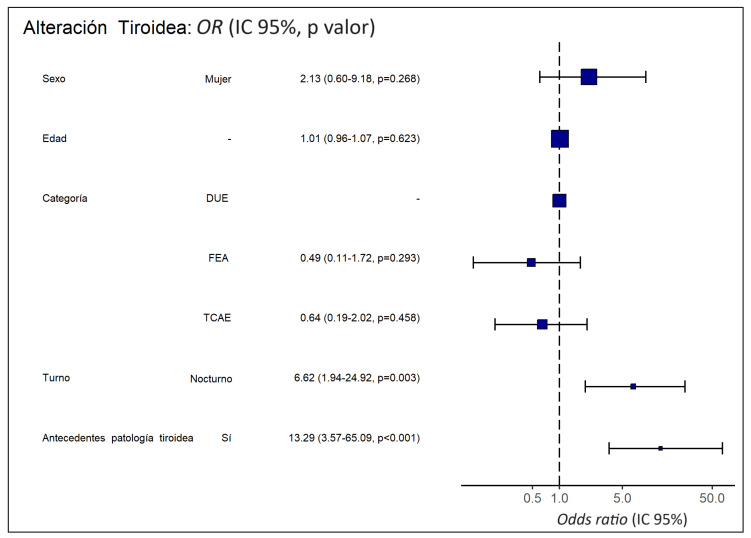
Diagrama de bosque del modelo multivariante de regresión logística (escala logarítmica).

OR: *odds ratio*; IC: intervalo de confianza; FEA: Facultativo Especialista de Área; DUE: Grado en Enfermería; TCAE: Técnico en Cuidados Auxiliares de Enfermería.

Se evaluó la asociación entre la presencia de alteraciones en la función tiroidea y las variables del estudio mediante logística. El modelo ajustado final muestra que trabajar de noches fijas y la presencia de antecedentes de patología tiroidea fueron predictores independientes de presentar alteraciones tiroideas (Tabla 4). La Figura 2 resume gráficamente el resultado del modelo multivariante final de regresión logística para predecir la presencia de alteraciones tiroideas en la muestra de personal estudiado.

**Tabla 4:** Asociación con la presencia de alteración de la función tiroidea

Variable	Alteración función tiroidea	Regresión logística			
		Univariante		Multivariante	
	n (%)	OR (IC95%)	p	OR (IC95%)	p
*Sexo*			0,144		0,268
Hombre	4 (15,4)	1		1	
Mujer	32 (29,9)	2,35 (0,82-8,50)		2,13 (0,60-9,18)	
*Edad (años), media (DE)*			0,997		0,623
Alteración sí/no	46,1 (10,1) vs 46,1 (9,8)	1,00 (0,96-1,04)		1,01 (0,96-1,07)	
*Categoría*					
DUE	21 (30,4)	1			
FEA	4 (14,8)	0,40 (0,11-1,19)	0,125	0,49 (0,11-1,72)	0,293
TCAE	11 (29,7)	0,97 (0,40-2,29)	0,940	0,64 (0,19-2,02)	0,458
*Turno nocturno*					0,003
No	26 (22,0)	1			
Sí	10 (66,7)	7,08 (2,31-24,46)	0,001	6,62 (1,94-24,92)	
*Antecedentes de patología tiroidea*					<0,001
No	25 (21,0)	1			
Sí	11 (78,6)	13,79 (3,96-64,47)	<0,001	13,29 (3,57-65,09)	

OR: *odds ratio*; IC: intervalo de confianza; DE: desviación estándar; DUE: Grado en Enfermería; TCAE: Técnico en Cuidados Auxiliares de Enfermería; FEA: Facultativo Especialista de Área.

## DISCUSIÓN

Este estudio intenta aportar una visión general sobre las posibles alteraciones que ejerce la alteración del ritmo circadiano, inducida por el trabajo a turnos y nocturno, en la función del eje HPT y en el desarrollo de enfermedades tiroideas.

Hemos observado que el 67% del personal del turno fijo nocturno presentó alteraciones de la función tiroidea, una cifra notablemente superior pero en concordancia con la obtenida por Burdelack y col, quienes describieron una prevalencia global de enfermedades tiroideas del 21,2% en una cohorte de 725 enfermeras y matronas con turnos nocturnos rotativos o fijos^15^. La prevalencia de alteraciones tiroideas observada en el grupo de trabajadores con noches fijas es claramente superior al 9,9% estimado por Valdés y col en la población española sana en 2017^2^.

Las alteraciones de la función tiroidea del personal del turno fijo nocturno correspondieron mayoritariamente a niveles altos de TSH y T4 normal. Los valores de TSH en sangre fueron significativamente superiores en el personal del turno de noche fijas respecto al del resto de turnos, incluidos los rotativos. Estos resultados concuerdan con diferentes estudios realizados en profesionales de la salud. Spiegel y col^16^ encontraron que el personal sanitario con periodo de privación del sueño nocturno presentaba niveles de TSH plasmática matutina más elevados respecto a sujetos con una noche de sueño normal. También Chang y col^12^ observaron que los niveles de TSH de una cohorte de enfermeras de la sala de agudos del hospital psiquiátrico más grande del sur de Taiwán eran significativamente más altos en quienes tenían restricción del sueño durante dos turnos nocturnos consecutivos en comparación con quienes habían estado libres de servicio durante al menos tres días. Moon y col^10^ observaron en el personal de un hospital universitario en Corea con turno de noche niveles de TSH significativamente más altos (0,303 mIU/L), tras a ajustar por edad y servicio, que en el resto del personal.

La media de TSH en sangre de nuestro estudio, aun encontrándose en el límite superior de la normalidad, duplicaba el observado en resto de turnos de trabajo, en línea con otro estudio realizado en sanitarios de origen hindú, incluido personal médico, enfermero, técnico y de apoyo, que describió valores medios de TSH significativamente más altos en el turno de noche (3,11mUI/L; DE: 1,81 vs 2,04 mUI/L; DE: 0,8)^17^. Diferentes estudios realizados en profesionales de la salud que han descrito que el trabajo nocturno podría relacionarse con niveles más altos de TSH en sangre, aunque dentro del rango fisiológico^14^^,^^15^. A pesar de que aún quedan por dilucidar los mecanismos responsables de tales trastornos hormonales, así como sus consecuencias fisiológicas, se puede inferir que los cambios que el turno de noche induce en el horario, el tiempo y la calidad del sueño podrían alterar el ritmo circadiano normal del cuerpo y conducir a una secreción circadiana anormal de TSH junto con otras alteraciones metabólicas.

Los niveles de tiroxina presentaron una distribución homogénea en los diferentes turnos de trabajo. Pese a la normal o fisiológica asociación negativa entre los valores de TSH y tiroxina (la hipofunción de la tiroides se compensa estimulando la secreción de TSH), en el personal con turno fijo nocturno dicha asociación fue más acusada, lo que podría hacer sospechar una mayor proporción de hipotiroidismo subclínico en dicho turno.

La elevación de niveles de TSH en sangre junto con niveles séricos de T4 dentro del rango fisiológico fue mucho más frecuente en mujeres (9,4 frente a 3,8%), en línea con el comportamiento epidemiológico del hipotiroidismo subclínico en la población española^17^.

En cuanto a las limitaciones del estudio, no se han tenido en cuenta variables individuales que podrían alterar los resultados obtenidos en el presente estudio son el origen étnico, la ingesta de calorías durante el turno de trabajo (posiblemente más elevada durante el turno de noche), el estado físico (incluidos el nivel de condición física y la actividad deportiva realizada antes/después del trabajo o antes de acostarse), así como el seguimiento de tratamientos indicados para comorbilidades que posiblemente influyan en los concentraciones séricas de TSH en sangre. Asimismo, cabe destacar que durante el proceso de recogida de datos surgieron algunos problemas que comprometieron al tamaño de muestra del estudio y favorecieron que existiera una distribución heterogénea en cuanto al número de trabajadores incluidos de cada turno. También la suspensión de exámenes de salud por la pandemia de COVID-19, la no inclusión de otros servicios del hospital con turnicidad, y la existencia de profesionales que declinaron participar en el estudio limitó el número de exámenes de salud realizados, lo que ha podido disminuir la fiabilidad de los resultados por falta de potencia. Tanto el pequeño tamaño muestral para el turno nocturno y para el sexo masculino, como el hecho de que la mayoría de personas con antecedentes de patología tiroidea y/o con turno nocturno sean mujeres, hace que no se haya podido evaluar si el sexo puede estar funcionando como una variable de confusión y/o modificadora de efecto. Tampoco se han considerado otros posibles factores de confusión que pudieran influir en la regulación de la función tiroidea, como hábitos tóxicos, tareas propias del turno de trabajo, tiempo de descanso durante los turnos o nivel de estrés.

En conclusión, trabajar en un turno fijo de noche se relacionó con concentraciones séricas de TSH más elevadas y con mayor frecuencia de alteraciones tiroideas que en el resto de turnos, generalmente niveles elevados de TSH con niveles de T4 dentro del rango fisiológico. La frecuencia de TSH elevada con tiroxina normal fue significativamente más frecuente en mujeres. Realizar un turno fijo nocturno y poseer antecedentes de alteración tiroidea fueron factores pronóstico de funcionamiento tiroideo alterado en este estudio.

Los protocolos específicos de vigilancia de la salud deberían incluir una evaluación de la función tiroidea mediante análisis de sangre al personal expuesto a turno nocturno que permita el diagnóstico precoz de alteraciones tiroideas. Además, permitiría evaluar condiciones particulares de hipersensibilidad que puedan requerir medidas preventivas especiales, a fin de proteger en la medida de lo posible la salud psico-física del personal expuesto, y minimizar la aparición de enfermedades o incluso de accidentes de trabajo.
